# Prevalence and spectrum of illness among hospitalized adults with malaria in Blantyre, Malawi

**DOI:** 10.1186/1475-2875-13-391

**Published:** 2014-10-02

**Authors:** Dalitso Segula, Anne P Frosch, Miguel SanJoaquin, Dalitso Taulo, Jacek Skarbinski, Don P Mathanga, Theresa J Allain, Malcolm Molyneux, Miriam K Laufer, Robert S Heyderman

**Affiliations:** Malawi-Liverpool-Wellcome Trust Clinical Research Programme, University of Malawi College of Medicine, P.O. Box 30096, Blantyre, Malawi; Department of Medicine, University of Malawi College of Medicine, Blantyre, Malawi; University of Minnesota School of Medicine, Minneapolis, MN USA; Malaria Branch, Centers for Disease Control and Prevention, Atlanta, GA USA; Malaria Alert Center, University of Malawi, College of Medicine, Blantyre, Malawi; Center for Vaccine Development, University of Maryland School of Medicine, Baltimore, MD USA; Liverpool School of Tropical Medicine, Liverpool, UK

**Keywords:** Malaria, Severe malaria, Prostration, Malawi, Rapid diagnostic tests, Hyperparasitaemia, Malaria epidemiology, Adult

## Abstract

**Background:**

As control interventions are rolled out, the burden of malaria may shift from young children to older children and adults as acquisition of immunity is slowed and persistence of immunity is short-lived. Data for malaria disease in adults are difficult to obtain because of co-morbid conditions and because parasitaemia may be asymptomatic. Regular surveys of adult admissions to a hospital in Malawi were conducted to characterize the clinical spectrum of malaria and to establish a baseline to monitor changes that occur in future.

**Methods:**

In 2011–2012, at Queen Elizabeth Hospital, Blantyre, four separated one-week surveys in the peak malaria transmission period (wet season) and three one-week surveys in the low transmission period (dry season) were conducted using rapid diagnostic tests (RDT) with confirmation of parasitaemia by microscopy. All adults (aged ≥15) being admitted to the adult medical wards regardless of the suspected diagnosis, were enrolled. Participants with a positive malaria test underwent a standardized physical examination and laboratory tests. Malaria syndromes were characterized by reviewing charts and laboratory results on discharge.

**Results:**

765 adult admissions were screened. 63 (8.2%) were RDT-positive with 61 (8.0%) positive by microscopy. Over the course of the seven study weeks, two patients were judged to have incidental parasitaemia, 31 (4.1%) had uncomplicated malaria and 28 (3.7%) had severe malaria. Both uncomplicated and severe malaria cases were more common in the rainy season than the dry season. Prostration (22/28 cases) and hyperparasitaemia (>250,000 parasites/μl) (9/28) were the most common features of severe malaria. Jaundice (4/28), severe anaemia (2/28), hyperlactataemia (2/28), shock (1/28) and haemoglobinuria (1/28) were less commonly seen, and no patient had severe metabolic derangement or organ failure. There were no deaths attributable to malaria.

**Conclusion:**

In this study of adults admitted to hospital in southern Malawi, an area with year-round transmission of *Plasmodium falciparum,* classical metabolic and organ complications of malaria were not encountered. Prostration and hyperparasitaemia were more common indicators of severity in patients admitted with malaria, none of whom died. These data will provide a baseline for monitoring trends in the frequency and clinical patterns of severe malaria in adults.

## Background

In Malawi, nearly six million suspected cases of malaria are treated annually [1]; the burden of malaria disease has been clinically observed to be disproportionately suffered by children. In malaria-endemic countries, this preponderance of malarial morbidity and mortality in children has been attributed to insufficient protective immunity [2]. Repeated exposure to malaria infection eventually leads to a state of semi-immunity, as a result of which older children and adults may be infected with malaria parasites, but have no or minimal symptoms [3]. However, in the context of changing epidemiology globally, recent research raises concern that the burden of malaria disease among adults may be underestimated, or may begin to increase, possibilities that have led to a renewed interest in the disease burden in this older population [4].

Like many countries in Africa, Malawi is scaling up malaria control interventions. Insecticide-treated net ownership was at 32% in urban and 10% in rural areas in 2000, but increased to 64% and 59% respectively by 2012 [5, 6]. The coverage of indoor residual spraying in urban areas has increased from 1.7% to 4.1% between 2010 and 2012 [1, 6]. In November 2007, artemisinin-based combination therapies were adopted as the recommended method of treatment and by 2012, 65% of children with fever who received anti-malarial treatment were given artemether-lumefantrine [6]. While expanded access to malaria control interventions is expected to reduce malaria morbidity and mortality in young children, there is a concern that a decrease in malaria transmission rates will delay the age at which clinical immunity is acquired, and that immunity will wane more rapidly owing to less frequent re-exposure, leading to greater susceptibility to disease among adults [2, 7, 8]. Without reliable data, collected in a standardized manner, it will be difficult to detect a shift in disease burden as a result of these public health interventions.

Routine surveillance is unlikely to be sensitive enough to detect changes in the burden of malaria among adults in most African settings. The diagnosis often relies on the clinical identification of malaria syndromes without laboratory confirmation; syndromic diagnosis alone will over-estimate the true incidence of parasitologically confirmed malaria [9], especially in an adult population with a high baseline rate of HIV infection [10] in which undifferentiated fevers are common. Routine testing for malaria infection among adults in highly malaria-endemic countries may also be difficult to interpret as adults may have multiple and complex co-morbidities, making problematic the interpretation of parasitaemia and its role in the causality of fever. In an outpatient setting in Malawi, 10% of HIV infected adults with a positive malaria test were found to have an additional clear cause of fever [11]. Prospective, systematic surveillance in adults for malaria disease is needed to determine the true burden in adults and to detect changes in that burden as malaria control improves.

To address this need, a prospective study was conducted using intensive periodic surveys to evaluate the prevalence of malaria infection and disease among hospitalized adults at a large teaching hospital in Blantyre, Malawi, and characterized their presentation, severity and outcome.

## Methods

### Study design

From February 2011 through January 2012, we conducted seven separate weeks of intensive malaria surveillance amongst adult (≥15 years) medical admissions to Queen Elizabeth Central Hospital (QECH) in Blantyre, Malawi. QECH is the district hospital for urban, peri-urban and rural population of about one million, and also serves as the referral hospital for the southern region of the country. The internal medical department has approximately 200 beds and admits 10–40 adults per day. During 2001–2010, microscopic parasitaemia rates among children presenting to QECH averaged approximately 25% [12]. At the time the study was being conducted, the hospital had adopted the 2010 WHO guidelines for treatment of Malaria [13]. Intravenous Quinine was used for severe malaria while artemether-lumefantrine oral combination therapy was used for uncomplicated malaria [6, 13].

Three surveillance weeks were undertaken during the dry season (April 2011-November 2011) and four during the rainy season (December 2011 – March 2012). During these surveillance periods all adults being admitted to the medical department (aged ≥15 years), regardless of the suspected diagnosis, were offered study enrolment. After informed consent, demographic data including age, sex and residence were collected. Residence was categorized as either rural or urban, and was defined as a place where a participant had spent a night or more two weeks prior to presentation. All participants were tested for malaria using a rapid diagnostic test [SD Bioline Malaria Ag Pf/Pan, a three band rapid diagnostic test (RDT) detecting *Plasmodium falciparum*-specific parasite lactate dehydrogenase and pan *Plasmodium*-specific pLDH, manufacturer’s sensitivity > 95.5%]. Thin and thick smears, conducted in a quality controlled research laboratory setting, were collected from all patients with a positive RDT for confirmation of infection and quantification of parasitaemia. A case of malaria infection was defined as a positive RDT confirmed by malaria smear microscopy.

All participants with positive RDTs underwent a standardized medical history and physical examination. Female participants were asked whether they thought they were pregnant. Measurements for full blood count, blood glucose, creatinine, bicarbonate, bilirubin and lactate were made. Blood cultures were collected in patients who had a temperature greater than 37.5°C or had reported a history of fever with the current illness; these cultures were processed according to standardized protocols [14].

Asymptomatic malaria infection was defined as RDT and blood smear positive without any fever, history of fever or other symptoms suggestive of malaria. Symptomatic malaria was defined as being present in any patient with a positive RDT and blood smear plus signs or symptoms consistent with malaria disease [13], with no alternative more likely cause of these symptoms or signs on chart review. Severe malaria was defined as symptomatic malaria with one or more of the WHO criteria for severe malaria [13]. For the purposes of this study we included prostration (inability to sit or stand unsupported) and hyperparasitaemia (>250,000 asexual parasites/μl) as indicators of severity [13], although these criteria have not been included in all versions of the criteria for severe malaria in adults. Uncomplicated malaria was defined as symptomatic malaria without any of the features of severe malaria. Fever was defined as a temperature of ≥ 37.5°C on admission.

A standardized review process was conducted to classify malaria infection cases as asymptomatic infection, uncomplicated malaria or severe malaria. Every study patient followed a standard predefined case report form (CRF). Clinical data were collected on enrolment using the CRF. Data were also extracted from the patients’ charts (review of patient charts) and from laboratory database using the predefined CRF. Two clinicians not involved in the patients’ day to day care independently reviewed the data, which included the clinical data and results of blood cultures and other laboratory investigations to determine if aetiology might be responsible for the symptoms associated with the current illness or if malaria was the most likely diagnosis.

### Statistical analysis

Statistical software SPSS version 21 (IBM) was used for data analysis. Descriptive statistics including proportions and medians are reported. Differences in proportions for categorical variables were evaluated using the Chi-squared test. Medians were compared by the Mann Whitney U test. Results were considered significant if p ≤ 0.05.

### Ethical review

The study was reviewed and approved by the University of Malawi College of Medicine Research and Ethics Committee and the University of Minnesota Institutional Review boards. Informed consent was obtained from all patients over 18 years and both informed consent from the guardian and assent from the participant were obtained for those between 15 and 18 years.

## Results

### Baseline characteristics

Between February 2011 and January 2012, a total of 769 patients were admitted to the adult internal medical wards at QECH during the three survey weeks in the dry season (April 2011-November 2011) and four survey weeks in the rainy season (December 2011 – March 2012). Almost all participants who were approached were enrolled in the study (765/769). Participant characteristics are described in Table 1. Most participants lived in the urban area of Blantyre. The HIV sero-status of the majority of patients screened was unknown (84%). Eleven percent were HIV sero-positive and 5% were HIV sero-negative. There were no women who reported that they were pregnant.

**Table 1:** **Demographics of adult admissions to Queen Elizabeth Hospital enrolled in study**

	Participants infected with malaria n=61	Participants not infected with malaria n=704	p-value*
Age (yrs); Median	28	35	<0.01
Gender			
Female, n (%)	43 (69)	397 (57)	0.1
Residence			
Urban, n (%)	39 (62)	502 (72)	0.6

*p-value for the difference between infected and non-infected participants.

Overall, 63/765 (8.2%) patients were RDT-positive. Sixty-one of the 63 RDT-positive patients (96.8%) had a positive malaria smear. *Plasmodium falciparum* was found by blood smear in all participants except one with *Plasmodium ovale* infection. The latter was negative for Pf-pLDH and positive for pan-LDH and was the only case with these findings. The overall prevalence of parasitaemia was 61/765 (8%) in the seven study weeks, but the distribution was highly seasonal. During the rainy season, approximately one out of every eight patients had malaria infection, compared to one in 30 during the dry season.

### Prevalence of asymptomatic, uncomplicated and severe malaria

Two (0.03%) of the participants screened had asymptomatic malaria infection. Uncomplicated malaria was found in 6.1% and 2.1% of admissions in the rainy and dry seasons respectively (p < 0.005). Severe malaria admissions were 6.1% in the rainy and 1.3% in the dry season (p < 0.0004). Among patients with malaria, the distribution of uncomplicated and severe disease was similar in both seasons. The single episode of *P. ovale* infection presented as uncomplicated malaria.

### Characteristics of severe malaria

**Figure 1:**
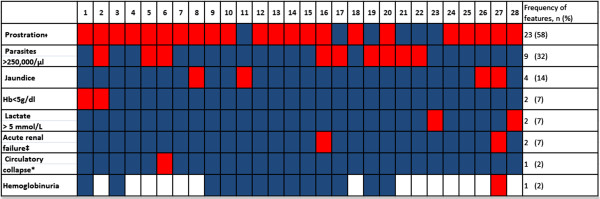
**Clinical features of 28 adults with severe malaria.** Each numbered column represents a patient with severe malaria. Presence of feature shown in red, Absence of feature shown in blue, Missing data in white. Hb, Haemoglobin; *systolic blood pressure less than 80 mmHg, ^‡^Unable to stand or sit without support, ^‡^Urine output < 400mls/24 hours despite adequate rehydration or creatinine ≥ 265 μmol/L.

The most common clinical finding of severe malaria was prostration, and the most common laboratory abnormality was hyperparasitaemia (Figure 1). There were no cases of cerebral malaria or hypoglycaemia detected. Severe anaemia and renal failure were uncommon (Figure 1). There was no difference between severe malaria syndromes of urban and rural residence. There were no deaths due to malaria disease amongst the participants enrolled in the seven intensive weeks. The average hospital stay was 2 days.

### Fever and parasitaemia

**Figure 2:**
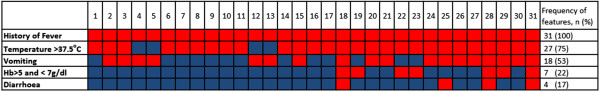
**Clinical features of 31 adults with uncomplicated malaria.** Each numbered column represents a patient with uncomplicated malaria. Presence of feature shown in red, Absence of feature shown in blue, Missing data in white. Hb, Haemoglobin.

Overall, 493/765 participants (64% of admissions) had fever (reported or measured pyrexia). Of participants reporting a history of fever or with pyrexia at presentation, 59/493 (12%) had symptomatic malaria - 46/257 (18%) in the rainy season, and 13/236 (6%) in the dry season. Of the participants with symptomatic malaria, 31/59 (53%) had uncomplicated malaria (Figure 2). The frequency of parasitaemia among those presenting with no pyrexia or history of fever was 2/272 (0.7%).

### Co-morbidities

In three participants with parasitaemia, other diagnoses were evident upon chart review. In two of these, there was no evidence of malaria disease (no fever or any other feature in keeping with malaria). One was known to have cryptococcal meningitis and came in for routine therapeutic lumbar puncture. The other participant had a stroke and no features of malaria. These two participants [2/765 (0.03%)] were categorized as having asymptomatic parasitaemia.

The third participant had pneumonia with evidence for concurrent severe malaria disease identified upon chart review. Features of malaria in this patient included pyrexia (39.2°C), hyperparasitaemia (418,200 parasites/μl of blood), thrombocytopaenia and severe anaemia. This participant was categorized as a severe malaria case with the additional diagnosis of pneumonia. All the 63 RDT positive participants had blood cultures done, and none was positive for a pathogen.

## Discussion

Malaria infection is a frequent cause of hospital admission among adults presenting to a large teaching hospital in Malawi, accounting for 8% of admissions; however the diagnosis of malaria only accounts for 12% of the causes of fever. This observation underlines the importance of parasitological diagnosis, if malaria is not to be overdiagnosed in this patient population. Use of a sensitive RDT allows for the slower and more laborious microscopy confirmation and quantification to be restricted to patients in whom it is likely to yield important additional information. The LDH-based RDT kits were highly specific (61/63, 98.6%).

A recent survey in several rural areas of Malawi reported decreasing prevalences of parasitaemia and anaemia in children aged <5 years (indicators used for monitoring effectiveness of preventive measures in the community); these changes were attributed to increasing use of insecticide-treated nets and a transition to artemisinin-based combination therapy [15]. It has yet to be seen whether such control measures will have an impact on malaria in adults. This sectional evaluation of malaria disease burden among adult hospital admissions provides a baseline for the evaluation of possible drivers of the burden of malaria disease in adults. It will be of special interest to monitor malaria disease trends in the coming years alongside data capturing the effectiveness of ongoing malaria control programmes.

A number of the classical syndromes of severe malaria were lacking in symptomatic adult patients in our study. A study conducted in KwaZulu-Natal, where malaria transmission is epidemic, found that renal failure and lactic acidosis were common features in addition to hyperparasitaemia among adults with severe malaria [16]. In contrast, severe malaria disease in our participants was characterized by hyperparasitaemia and prostration, and the life-threatening syndromes often found in adults in low-transmission areas – acute kidney injury, pulmonary oedema/ARDS, disseminated intravascular coagulation, coma and convulsions – were unusual or absent, as were syndromes that are common in children in Malawi, including severe anaemia, lactic acidosis, hypoglycaemia and cerebral malaria. In the same setting and during the same period as this study, cerebral malaria and severe malarial anaemia were common causes of admissions to the paediatric wards [12]. This familiar difference between children and adults in a high-transmission area is likely to be due to the gradual acquisition of partial immunity to *P falciparum* in endemic areas [2, 16–19].

The lack of classical severe malaria syndromes these patients has important implications for the surveillance of malaria, considering that many healthcare facilities with limited resources throughout sub-Saharan Africa often rely on clinical presentation and the ascertainment of coma, seizures and renal failure for severe malaria diagnosis and management decisions in adults [9, 20]. The data confirm the emphasis of WHO that reliance on clinical features alone for the diagnosis of severe malaria is inaccurate [13]. Prostration (extreme weakness) is a non-specific syndrome with many causes other than malaria, and a diagnosis of severe malaria may be missed if a parasitological test is not done; conversely, a parasitological test is needed to reduce overdiagnosis of malaria in febrile patients.

As RDTs are rolled out in Malawi, there is a possibility that smears may not be done routinely and that hyperparasitaemia may be under-ascertained. It is not clear if hyperparasitaemia is an important marker of severity among semi-immune adults. In order to detect this sign of severe disease and to monitor any changes in its incidence, hospitalized adults with a positive RDT should undergo quantification with smears as the standard of care.

This study was limited by the cross-sectional nature of the data collection and the fact that it was conducted in a single urban centre within a single year. Although it may not be generalizable to smaller hospitals or referral centres in other transmission settings, a valid baseline has been established by which to compare future studies and highlight some of the key features of adult malaria in malaria-endemic settings. Although many participants in this study had features that traditionally define severe malaria, including prostration and hyperparasitaemia, yet organ failures and severe metabolic derangements were rare, there were no deaths and hospitalization was short (mean two days). Further monitoring should include all of these indicators of malaria severity, in order to identify any changes in the incidence or pattern of the malaria burden among adults as control measures are increasingly deployed.

## Conclusion

Uncomplicated and severe malaria disease among adults admitted to a hospital in Malawi has been described using definitions based on the WHO guidelines. Prostration and hyperparasitaemia were the commonest indicators of severe malaria, and many of the classical syndromes seen in adults in low-transmission areas were not observed. Failure to consider malaria as one of the possible causes of prostration would have underestimated the burden of severe malaria. Surveillance of severe malaria disease in adults should be based on both clinical assessment and parasitological tests, with microscopy for confirmation and quantification of parasitaemia.
